# Study on the Correlation between Life Expectancy and the Ecological Environment around the Cities along the Belt and Road

**DOI:** 10.3390/ijerph20032147

**Published:** 2023-01-25

**Authors:** Chang Li, Jing Wu, Dehua Li, Yan Jiang, Yijin Wu

**Affiliations:** Key Laboratory for Geographical Process Analysis & Simulation of Hubei Province, College of Urban and Environmental Science, Central China Normal University, Wuhan 430079, China

**Keywords:** belt and road, life expectancy, ecological environment, spatiotemporal lag spatial cross-correlation analysis, comprehensive ecological index

## Abstract

The impact of building the Belt and Road on the ecological environment and the health of the related cities along this belt deserves more attention. Currently, there are few relevant pieces of research in this area, and the problem of a time lag between the ecological environment and health (e.g., life expectancy, LE) has not been explored. This paper investigates the aforementioned problem based on five ecological indicators, i.e., normalized difference vegetation index, leaf area index, gross primary production (GPP), land surface temperature (LST), and wet, which were obtained from MODIS satellite remote-sensing products in 2010, 2015, and 2020. The research steps are as follows: firstly, a comprehensive ecological index (CEI) of the areas along the Belt and Road was calculated based on the principle of component analysis; secondly, the changes in the trends of the five ecological indicators and the CEI in the research area in the past 11 years were calculated by using the trend degree analysis method; then, the distributions of the cold and hot spots of each index in the research area were calculated via cold and hot spot analysis; finally, the time lag relationship between LE and the ecological environment was explored by using the proposed spatiotemporal lag spatial crosscorrelation analysis. The experimental results show that ① there is a positive correlation between LE and ecological environment quality in the study area; ② the ecological environment has a lagging impact on LE, and the impact of ecological indicators in 2010 on LE in 2020 is greater than that in 2015; ③ among the ecological indicators, GPP has the highest impact on LE, while LST and Wet have a negative correlation with LE.

## 1. Introduction

The “Belt and Road” (B&R), which is the abbreviation of the Silk Road Economic Belt and the Maritime Silk Road, was proposed and launched in 2013. It connects China with raw material sources in the global market and greatly affects the future of global trade, especially in Asia, Europe, and Africa [1,2]. The B&R has built a series of infrastructures, including railway lines, highways, port facilities, and energy pipelines across the continent [3], along with the development of ports along the Pacific and Indian oceans [1]. The construction of this infrastructure has promoted the orderly flow of economic elements, the efficient allocation of resources, and the deep integration of markets, and gradually, has formed regional co-operation from point to area and from line to piece. The ecological environment of the B&R is crucial to China. However, the rapid development of the economy and the construction of infrastructure has put pressure on the ecological environment of the B&R and may affect the health of the residents in the region.

Health is the core of human development, the foundation of social development [4], and the important background of the B&R. In essence, health also represents the premise for the development of productive forces, and the continuous development of society requires healthy physiques [5,6]. Thus, the economy and health affect each other [7]. The construction of a healthy China will be of great significance to economic development, social stability, and people’s life [8]. With the rapid development of the social economy, the global ecological environment has been damaged to varying degrees, and the quality of the ecological environment has become an important factor affecting human health. For example, as a significant environmental risk threatening human health, air pollution has an important impact on the public health of cities in the Yangtze River Economic Belt [9]. Besides, global warming and precipitation caused by climate change are also posing an increasing health risk by affecting the incidence rate of human diseases [10]. With the increasingly prominent contradiction between man and nature, ecological and environmental issues, such as “double carbon” [11,12,13], “global warming” [14,15], and glacier melting [16,17], have become the focus of public, social, academic, and international attention in promoting the sustainable development of human society. However, the B&R has destroyed the original ecological environment and reduced biodiversity, along with the large-scale deforestation of infrastructure construction. The green, shared, and sustainable development of the B&R and the promotion of the construction of ecological civilization have become important measures to promote the construction of a community of human and natural life, deal with climate change and maintain global ecological security as well as promoting the healthy development of mankind. Therefore, it is necessary to further study the relationship between the ecological environment and the health of residents along the B&R.

Life expectancy (LE) is an important indicator to measure the health level of national, ethnic, and regional residents. Compared with child mortality, maternal mortality, and other indicators, LE can better reflect the mortality of the population regarding overall age [18]. The ecological environment is one of the important factors affecting LE [19]. Gulis et al. used LE as an evaluation index to measure the health of the ecological environment [20]. At present, research on the relationship between LE and the ecological environment tends to focus on the impact of various environmental factors on LE. For example, Nkalu et al. used the GARCH model to study the impact of the environment on LE in Africa [21]. Wang et al. found that economic growth and a reduction in air pollution can promote the growth of LE [19]; Wu et al. used the linear mixed effects model to analyze PM2.5 and LE and found that a decrease in PM2.5 was related to an increase in LE, and the strict implementation of the Action Plan for Air Pollution Prevention and Control had significant benefits for LE [22]; however, because the LE in each period is the average of the past LE, the LE index is lagging behind. Some scholars try to study the time lag and spatial lag effects of environmental factors on LE. In terms of a time lag, Cheng et al. used the distributed nonlinear lag model to fit the time relationship between air pollution and LE [23]; Muhammad Haroon Shah et al. used the nonlinear autoregressive distribution lag model to reveal the long-term relationship among environmental quality, public environmental expenditure, and LE [24]; In terms of spatial lag, Ladoy et al. found that neighborhood characteristics have an impact on the spatial lag of LE in a study area [25]. However, at present, studies on the combination of time lag, spatial lag, and LE have not been reported.

With the national emphasis on environmental health, research on the relationship between environment and health in China have gradually increased, but most of the work focuses on the impact of various types of pollution on health. For example, Liu et al. studied the impact of air pollution on Chinese public health [26]; Yang et al. used the spatial econometric model to analyze the impact of air pollution, economic development, and other factors on public health in the cities of the Yangtze River Economic Belt. The results showed that there was a significant spatial correlation between air pollution and public health in the cities of the Yangtze River Economic Belt [9]; Xiao et al. studied the potential risk of heavy metal pollution on the health of residents in Hanzhong City, Shaanxi Province [27]; Zhang et al. studied the harm of mineral mining pollution on human health in China [28]. However, there are few studies on the relationship between the environment and health in mainland China under the B&R.

With the development of remote-sensing technology, the indicators and methods of ecological environment quality evaluation are constantly improving and perfecting. The establishment of the indicator system is the key to using it for evaluation. Assessing environmental quality is a complex and challenging task. In 2006, the Technical Specifications for Ecological Environment Assessment (Trial) issued by the Ministry of Environment of the People’s Republic of China stipulated the indicator system for ecological environment status assessment and the calculation methods for each indicator, including biological abundance index [29], land surface temperature (LST) [30], plant coverage index, and other indicators. These indicators only reflect the ecological quality from a single aspect, such as soil and vegetation. However, the interaction between each environmental component in the ecosystem will affect the entire ecological environment. Therefore, it is best to use comprehensive, ecological-environment-quality indicators for this type of evaluation [27,31,32]. For example, Zhang et al. selected five indicators, i.e., impermeable surface, normalized difference vegetation index, land surface temperature, greenness, and brightness, and constructed comprehensive evaluation factors through a factor analysis method [33]; Ouyang et al. selected the normalized difference vegetation index (NDVI), water use efficiency (WUE), and leaf area index (LAI) to calculate weight using the projection pursuit model (MEQ-PPM) to obtain the comprehensive ecological quality index [34]; Li et al. used the analytic hierarchy process (AHP) to study the ecological quality evaluation of hilly areas with red soil by integrating nine factors, including water resources and vegetation coverage [35]. However, for the land area of China under the B&R, there has been no relevant research to select comprehensive evaluation indicators regarding the ecological environment.

To sum up, this current research area needs to be improved in the following aspects:

(1) For research on LE, the spatiotemporal lag is not fully considered, and the results deviate from reality. The impact of related factors on LE should be analyzed by combining time lag and spatial lag;
(2) In terms of research on the relationship between the ecological environment and health, research on the ecological environment and LE of China’s land area in the B&R has not yet been reported;
(3) There are few studies on the ecological environment indicators of China’s land area in the B&R and few studies on the comprehensive ecological indicators.

In view of the shortcomings of the current research, this paper has the following innovations and contributions:

(1) A time lag spatial crosscorrelation analysis method for the ecological environment and LE of the B&R is proposed for the first time. In this paper, the influence of the ecological environment on the time lag and spatial lag of LE is considered;
(2) This paper reveals the spatial and temporal patterns and laws of the ecological environment and LE of the B&R for the first time. At present, there are not many reports on cities along the B&R in China, and there are few studies on LE in the cities along China’s land area around the B&R. Most of them only focus on the construction of a healthy B&R;
(3) This paper provides a new objective assessment method for the ecological assessment of the B&R area. A remote-sensing comprehensive ecological index (RSCEI) is proposed. Five indicators, i.e., land surface temperature, gross primary productivity, leaf area index, fractional vegetation cover, and ground humidity, were selected to be used for the principal component analysis (PCA) to regress and integrate the comprehensive indicators of the ecological environment of the B&R region during 2010, 2015, and 2020 for an ecological quality assessment, which is more advantageous than a single ecological index.

## 2. Study Area and Data Preprocessing

### 2.1. Scope and Overview of the Study Area

**Figure 1:**
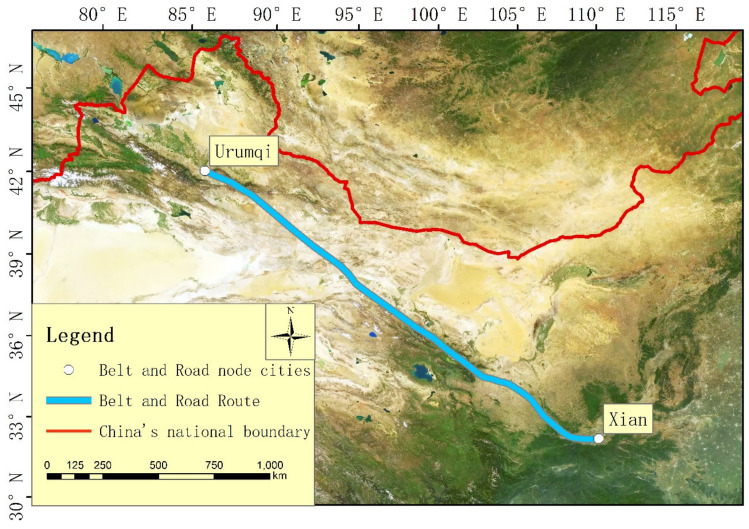
Overview of the Belt and Road Initiative.

**Figure 2:**
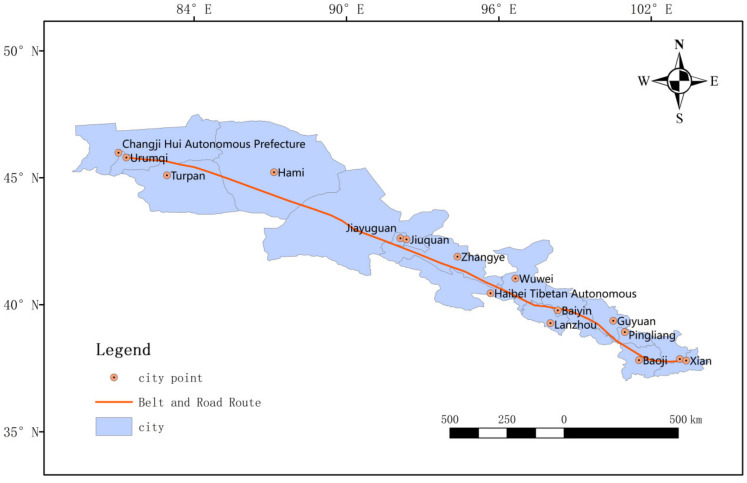
Overview of the study area.

This paper takes the “Silk Road Economic Belt” from Xi’an to Urumqi in the Chinese Mainland, which is located in northwest China. As seen in Figure 1, the study area is located at 85°17′ E~109°49′ E, 33°42′ N~45° N, with an area of about 604,518.13 km^2^. It passes through five provinces or autonomous regions, including Shaanxi Province, Gansu Province, Ningxia Province, Qinghai Province, and Xinjiang Uygur Autonomous Region. There are 16 cities or prefectures, i.e., Xi’an City, Xianyang City, Baoji City, Pingliang City, Guyuan City, Lanzhou City, Baiyin City, Wuwei City, Zhangye City, Jiayuguan City, Jiuquan City, Haibei Tibetan Autonomous Prefecture, Urumqi City, Turpan City, Hami City, and Changji Hui Autonomous Prefecture, which can be seen in Figure 2.

The study area has a temperate continental climate, with mostly arid and semiarid areas. In winter, it is cold and has little rainfall. In summer, it is hot and dry. The temperature in the hottest month is above 20 °C, and the temperature in the coldest month is below 0 °C. The annual average temperature from 2010 to 2020 is about 11.01 °C. From 2010 to 2020, the average temperature in winter was about −1.3 °C; the average temperature in spring was about 12.6 °C; the average temperature in summer was about 21.7 °C, and the average temperature in autumn was about 11.5 °C. Besides, the annual average precipitation from 2010 to 2020 was 130~330 mm. From 2010 to 2020, the average precipitation in winter was about 15.15~37.56 mm; the average precipitation in spring was about 173.25~220.5 mm; the average precipitation in summer was about 185.5–268.34 mm, and the average precipitation in autumn was about 104.11~167.39 mm. The terrain there is mainly plateau, mountain, and basin. The eastern part mainly consists of the Inner Mongolia Plateau and the Loess Plateau, while the western part is alternately distributed with mountains and basins, such as Qilian Mountains, Tianshan Mountains, Turpan Basin, and Junggar Basin. The vegetation there is mainly grassland and desert. The eastern plateau is mainly grassland and gradually transits to desert grassland and desert to the west. A large area of desert is distributed in the western basin. Due to the arid climate, there are few rivers, and most of them are seasonal rivers and mainly inland rivers and inland lakes. There is lots of sandy soil. Affected by natural conditions, people there mainly develop oasis agriculture, irrigated agriculture, and animal husbandry. Transportation is relatively convenient, including expressways, railways, subways, aviation, pipelines, and other transportation modes. Coal, oil, natural gas, and other mineral resources are abundant, and the potential for energy development is huge. The culture has distinct characteristics, such as regionality, nationality, and pluralism.

### 2.2. Data Sources

#### 2.2.1. MODIS Remote-Sensing Data

MODIS, a medium-resolution imaging spectrometer mounted on the Terra and Aqua satellites, can scan 2330 km (wide) and obtain global observation data every 1~2 days. Due to its advantages of a wide spectral range, simple data reception, and high update frequency, it is widely used in large-scale ecological research. All indicator data are derived from MODIS remote-sensing products (https://ladsweb.modaps.eosdis.nasa.gov/, accessed on 7 December 2022). Specific data are shown in Table 1.

The above MODIS data products, obtained in 2010, 2015, and 2020, are of a high grade and have been corrected for by radiation and atmosphere.

Table 2 shows the number of pixels, observations, mean, standard deviations, minimums, and maximums of GPP, LAI, LST, NDVI, and WET. The number of pixels is the total number of pixels in 16 county areas for each indicator; NDVI is observed once every 16 days, and GPP, LAI, LST, and WET are observed once every 8 days, so the number of observations in a year is 23 or 46, and the product of the total number of observed counties was the total number of observations, i.e., 46 × 16 = 736, and 23 × 16 = 368; the mean, standard deviation, maximum, and minimum are the total values of each indicator in 16 counties per year. (Note: GPP, LAI, LST, and NDVI are abbreviations for gross primary production, leaf area index, land surface temperature, and normalized difference vegetation index, respectively).

#### 2.2.2. Life Expectancy Data

This paper selects the LE data from 2010, 2015, and 2020, as shown in Table 3. Most of the data come from the statistical yearbooks of the cities or states and the data published on the official website of the local government. Some unpublished data were obtained by the interpolation method in this study.

### 2.3. Data Preprocessing

In this paper, the MODIS remote-sensing data were preprocessed. LE data were missing for some years in some areas. The life expectancy data from Baiyin City, Baoji City, Changji Hui Autonomous Prefecture, Hami City, Jiayuguan City, Wuwei City, Xianyang City, and Zhangye City in 2010, Changji Hui Autonomous Prefecture, Haibei Tibetan Autonomous Prefecture, and Zhangye City in 2015 and Changji Hui Autonomous Prefecture in 2020 are missing, and the missing data were obtained by interpolation.

## 3. Methodology

**Figure 3:**
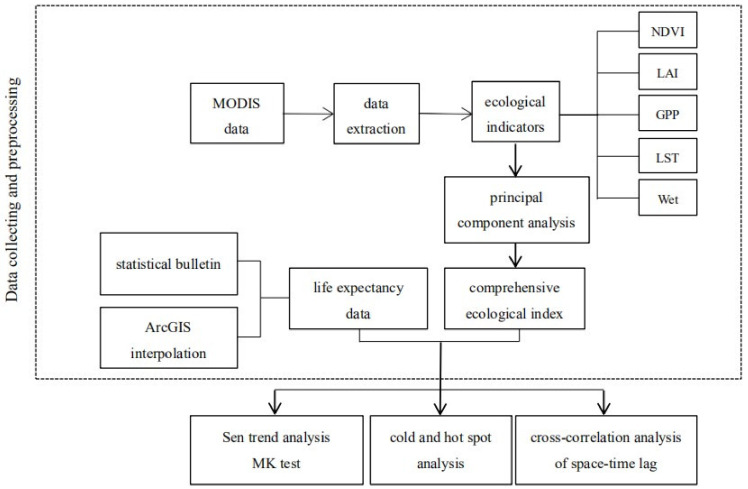
Research technical roadmap.

The technical roadmap of this study is shown in Figure 3.

### 3.1. Construction of the Ecological Indicators

(1) Normalized difference vegetation index (NDVI)

Normalized difference vegetation index (NDVI) [36] is a reference index to reflect the vegetation growth in the region.

(2) Leaf area index (LAI)

LAI [37] is an important structural parameter of ecosystems, which can provide structured quantitative information.

(3) Gross Primary Production (GPP)

Gross Primary Production (GPP) [38], also called the gross primary productive force, refers to the amount of organic carbon fixed by green plants through photosynthesis per unit of time.

(4) Land surface temperature (LST)

Land surface temperature (LST) is a key factor in surface physical processes at regional and global scales [39]. LST is also an important parameter with which to study the material exchange and energy exchange between the surface and atmosphere. The gray value obtained from the MODIS data is converted into degrees Celsius [40].

(5) Wet

The Wet index is also an important factor affecting vegetation growth. Tasseled Cap Transformation (TCT) is used for orthogonal linear transforms of multibands based on the statistical characteristics, which is often used for the compression and redundancy removal of remote-sensing data. The Wet index adopted in this paper was obtained by the TCT method [41].

### 3.2. Calculation of Comprehensive Ecological Score 

In this study, the above-mentioned five ecological indicators were transformed to a CEI as *P*, based on principal component analysis (PCA). The PCA method can convert multiple indicators (with correlations) into independent indicators and reduce their dimensions. The specific steps are as follows:

(1) First, standardize the data, i.e., normalize the data;
(2) Second, establish the correlation coefficient matrix of each index;
(3) Third, calculate the eigenvalues and corresponding eigenvectors;
(4) Fourth, calculate the variance contribution rate and determine the number of principal components, *k*;
(5) Finally, carry out the weighted summation of the k principal components to obtain the CEI as *P*, whose expression is Formula (2).

The biggest advantage of the PCA method is that it can get objective, comprehensive scores driven by data without subjective evaluations, such as the analytic hierarchy process (AHP) and Delphi method.

The following formula shows the relationship between each principal component and each index:

(1) $$\left(\begin{array}{c} X_{1}\\ X_{2}\\ X_{3}\\ \cdots \\ X_{5} \end{array}\right)=\left(\begin{array}{ccccc} \phi_{11} & \phi_{12} & \phi_{13} & \phi_{14} & \phi_{15}\\ \phi_{21} & \phi_{22} & \phi_{23} & \phi_{24} & \phi_{25}\\ \phi_{31} & \phi_{32} & \phi_{33} & \phi_{34} & \phi_{35}\\ \cdots & \cdots & \cdots & \cdots & \cdots \\ \phi_{51} & \phi_{52} & \phi_{53} & \phi_{54} & \phi_{55} \end{array}\right)\left(\begin{array}{c} \mathrm{NDVI}\\ \mathrm{LAI}\\ \mathrm{GPP}\\ \mathrm{LST}\\ \mathrm{Wet} \end{array}\right)$$

The first *n* principal components whose cumulative variance contribution rate exceeds 80% are taken as the principal components of the final ecological comprehensive index. Then, the *P* is calculated, whose expression is

(2) $$P=\sum_{i=1}^{n}k_{i}\times X_{i}$$

where *k_i_* (*i* = 1, 2, 3, …, *n*) represents the variance contribution rate of the *i*-th principal component, *n* represents the number of principal components, and *X_i_* represents its corresponding *i*-th principal component. 

### 3.3. Trend Analysis

The Theil-Sen median method, also called Sen slope estimation, is a robust nonparametric statistical trend calculation method. This method is efficient and insensitive to measurement errors and outlier data. This method is often used in the trend analyses of long-term series data. The specific formula is as follows:

(3) $$\beta =\mathrm{Median}\left(\frac{x_{j}-x_{i}}{j-1}\right),j>i,i=1,2,3,\ldots ,n$$

where *x_j_* and *x_i_* are the time series elements of the trend to be analyzed. *β* is the slope of the regression equation, i.e., the change trend of the variable. When *β* > 0, the variable shows an upward trend. When *β* < 0, the variable shows a downward trend.

The Mann–Kendall test method is a nonparametric test method. Compared with other parametric test methods, this method does not require samples to follow a certain distribution. Besides, it is less interfered with by outliers and is more suitable for sequential variables. The specific formula is as follows, where *S* is the statistic and *Z* is the test statistic. When *Z* is positive, it indicates that the data tend to increase with time. When *Z* is negative, it indicates that the data tend to decrease with time. A value of *Z* = 0 indicates that the data have no tendency to increase or decrease.

(4) $$S=\sum_{i=1}^{n-1}\sum_{j=i+1}^{n}\mathrm{sign}\left(x_{j}-x_{i}\right)\begin{array}{c} \;\;\;\;\;\;\;\;\;\;\left(i=2,3,\ldots ,n\right) \end{array}$$

(5) $$Z=\left\{\begin{array}{c} \left(S-1\right)/\sqrt{n\left(n-1\right)\left(2n+5\right)/18}\\ 0\\ \left(S+1\right)/\sqrt{n\left(n-1\right)\left(2n+5\right)/18} \end{array}\right.\begin{array}{c} \;\;\;\;\;S>0\\ \;\;\;\;\;S=0\\ \;\;\;\;\;S<0 \end{array}$$

### 3.4. Cold and Hot Spot Analysis

Cold and hot spot analysis is based on the idea of the zero hypothesis tests commonly used in statistical inference. The goal of cold and hot spot analysis is to identify regions with statistical significance clustering, indicating that there is a spatial correlation. The formula is

(6) $$G^{*}{}_{i}=\frac{\sum_{j=1}^{t}w_{i,\;\;\;,}x_{j}-\bar{X}\sum_{j=1}^{t}w_{i,\;\;\;,}}{\sqrt[{s}]{\left[t\sum_{j=1}^{t}w_{i,\;\;\;,}^{2}-{\left(\sum_{j=1}^{t}w_{,\;\;\;,}\right)}^{2}\right]/\left(t-1\right)}}$$

where *x_j_* is the attribute of the spatial until *j*, e.g., NDVI; *w_i,j_* represents the spatial weight between spatial units *i* and *j*; *t* is the number of spatial units; $\bar{X\;}$ is the mean value; *S* is the standard deviation; The statistical result obtained by G*_i_ is *z* score. Its significance is shown as follows: when *z* > 0, it indicates hot spots, i.e., hot spots gather more closely; when *z* < 0, it indicates cold spots, i.e., cold spots gather more closely.

### 3.5. Time Lag Spatial Crosscorrelation Analysis

#### 3.5.1. Global Crosscorrelation Analysis

Global crosscorrelation analysis is used to explore the overall spatial distribution among different attribute variables. Its calculation formula is as follows:

(7) $$I_{global}=\frac{n\sum_{i=1}^{n}\sum_{j=1}^{n}w_{ij}[{(\mathrm{LE})}_{i}^{\left(t\right)}-(\bar{\mathrm{LE}}{)][(\mathrm{CEI})}_{i}^{\left(t-m\right)}-(\bar{\mathrm{CEI}})]}{\sum_{i=1}^{n}\sum_{j=1}^{n}w_{ij}\sqrt{\sum_{i=1}^{n}{[{(\mathrm{LE})}_{i}^{\left(t\right)}-(\bar{\mathrm{LE}})]}^{2}{{\left[(\mathrm{CEI}\right)}_{i}^{\left(t-m\right)}-(\bar{\mathrm{CEI}})]}^{2}}}$$

where CEI is the comprehensive ecological index; LE is life expectancy; (*t*) means the *t*-th time point; (*t−m*) represents the (*t−m*)-th time point, reflecting the time lag.

#### 3.5.2. Local Crosscorrelation Analysis

Local crosscorrelation analysis can explore the correlation between CEIs and LE in local regions. Its calculation formula is as follows:

(8) $$I_{local}=\frac{n[{\left(\mathrm{LE}\right)}_{i}^{\left(t\right)}-\bar{\mathrm{LE}}]\sum_{j}w_{ij}[{(\mathrm{CEI})}_{i}^{\left(t-m\right)}-\bar{\mathrm{CEI}}]}{\sqrt{\sum_{i}{\left[{\left(\mathrm{LE}\right)}_{i}^{\left(t\right)}-\bar{\mathrm{LE}}\right]}^{2}{\left[{(\mathrm{CEI})}_{j}^{\left(t-m\right)}-\bar{\mathrm{CEI}}\right]}^{2}}}$$

where CEI is the comprehensive ecological index; LE is life expectancy; (*t*) and (*t−m*) have the same meanings as above.

## 4. Results and Analysis

### 4.1. CEIs

**Figure 4:**
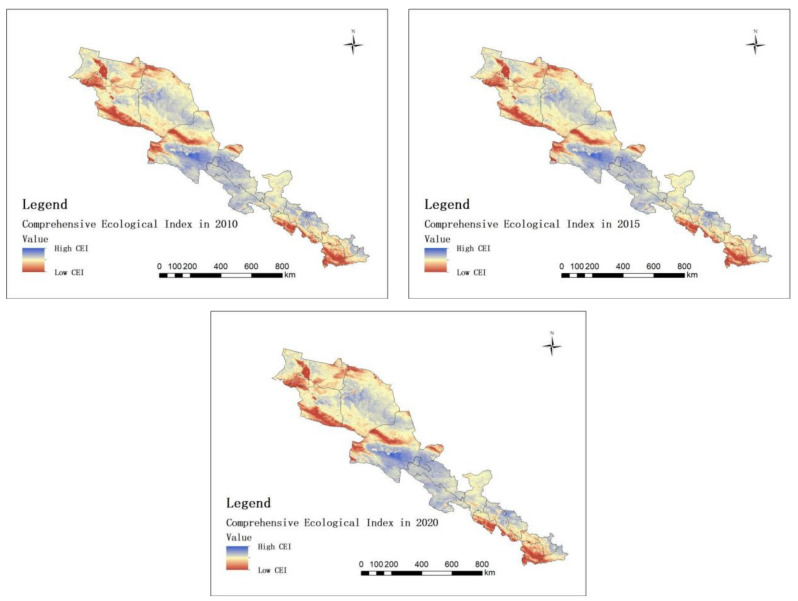
Distribution of CEI in 2010, 2015, and 2020.

The five indicators (NDVI, LAI, GPP, LST, and Wet) of the study area in 2010, 2015, and 2020 were obtained, respectively. Then, the PCA (Table 1) was carried out, and the distribution results of the CEIs in 2010, 2015, and 2020 are shown in Figure 4.

Table 4 shows that, in the first principal component (PC1), the indicators with larger coefficients are GPP, LAI, and NDVI, and the indicators in the three periods are positive and large. In the second principal component (PC2), LST is the index with larger coefficients in the first two periods: 0.955 and 0.984 in the two periods, respectively, while NDVI is the index with a larger coefficient in the third period: 0.709. Since the cumulative variance contribution rate of the first two principal components of the data in 2020 did not reach 80%, the first three principal components were selected. In the third principal component (PC3), LST has a larger coefficient of 0.787.

Figure 4 shows that the CEIs in the middle of the study area are high, i.e., Jiuquan City, Jiayuguan City, Zhangye City, and Haibei Tibetan Autonomous Prefecture. However, the CEIs in the southeast and northwest of the study area are low, i.e., Lanzhou City, Baoji City, Pingliang City, Urumqi City, Turpan City, and Changji Hui Autonomous Prefecture.

### 4.2. Correlation Analysis

#### 4.2.1. Correlation Analysis Results

Without considering the spatial distribution, the correlation between the annual index data and LE (Table 4) and the time lag correlation (Table 5) were calculated. It can be seen from the table that most of the index data are positively correlated with LE. The correlation between annual LST and the corresponding LE is small or negative; LST and NDVI in 2020 are negatively correlated with LE in 2020. Through spatiotemporal lag analysis, it was found that LST and Wet in 2010 and 2015 are negatively correlated with LE in 2015 and 2020, respectively.

#### 4.2.2. Spatial Correlation Analysis

##### Trend Analysis

**Figure 5:**
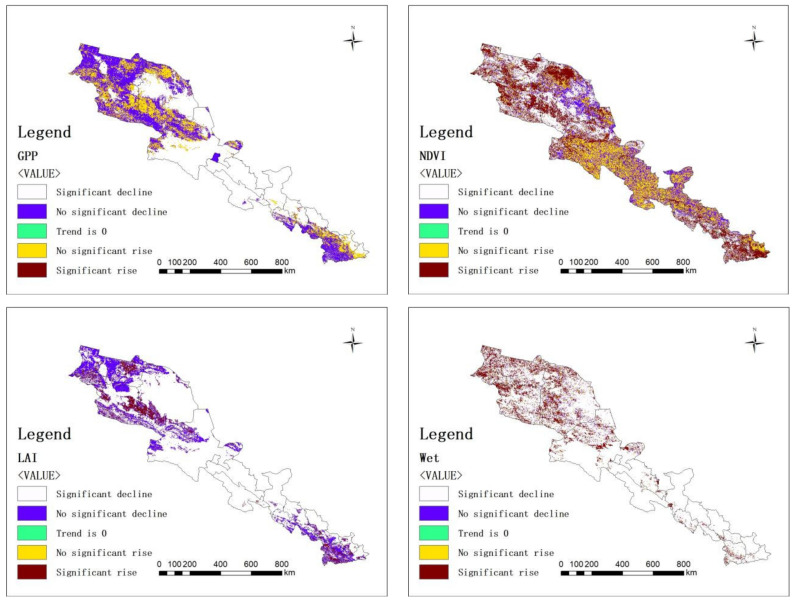

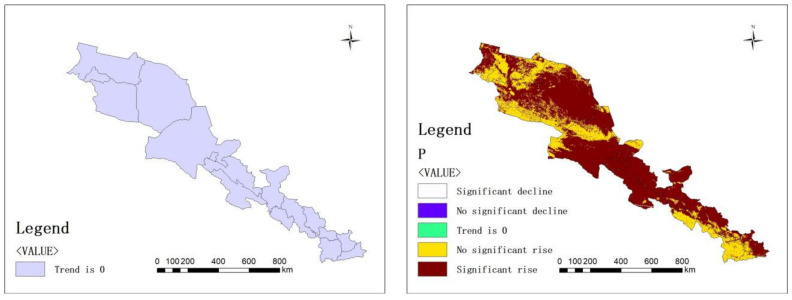
Result chart of the trend degree for each index dataset.

NDVI, LAI, GPP, LST, Wet, and CEI were obtained through trend degree analysis and MK testing. The trend degree results of the six indicators are shown in Figure 5. It can be seen that the trend degree for vegetation growth in the study area is basically rising; LAI and GPP decreased in the middle of the study area and decreased or increased slightly in the southeast and northwest of the study area; Wet has an obvious upward trend in the northwest of the study area; the trend for LST in the study area is almost zero. In general, the CEI in the study area shows an upward trend.

##### Spatial Cross-Correlation Analysis

**Figure 6:**
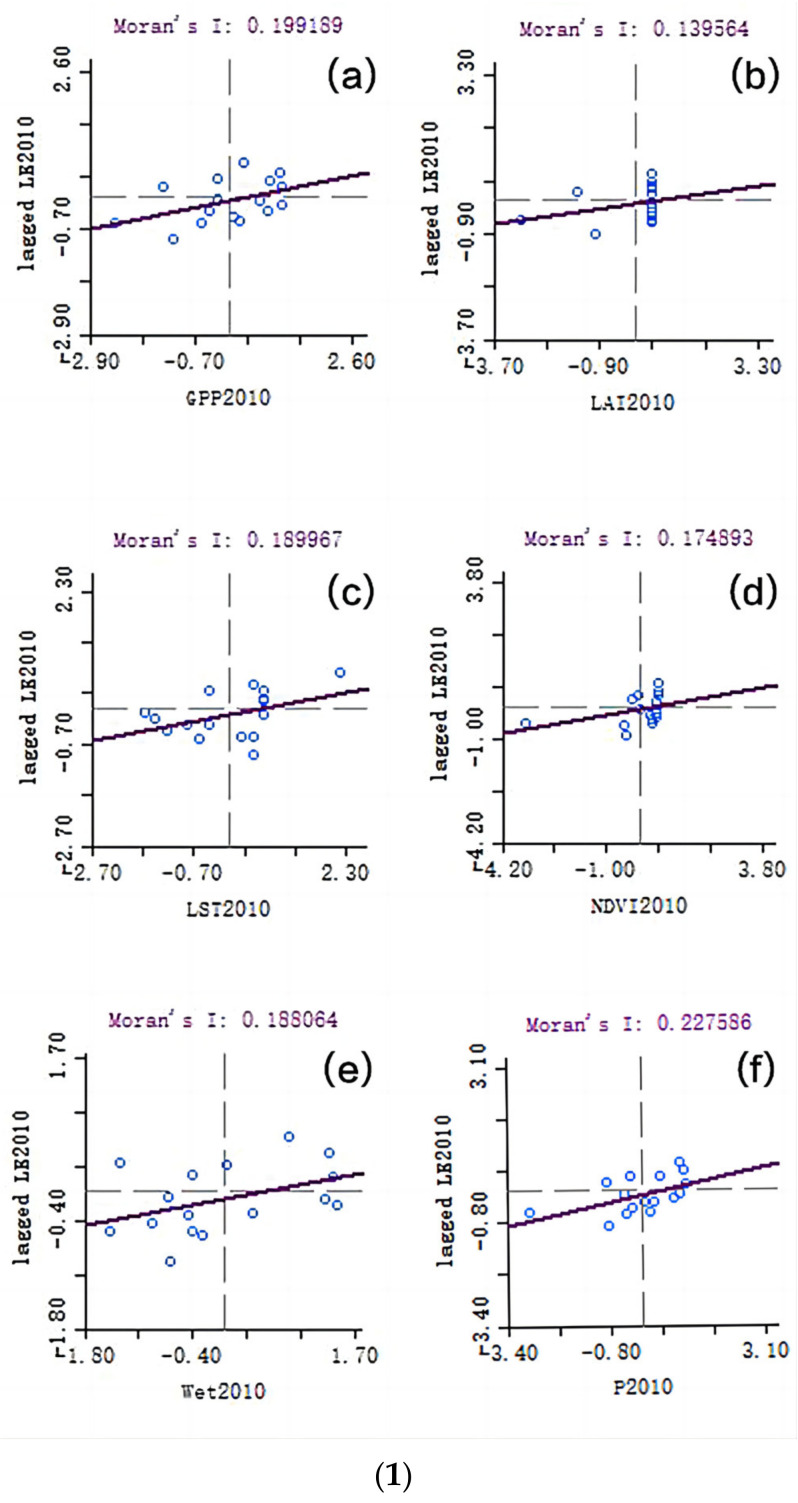

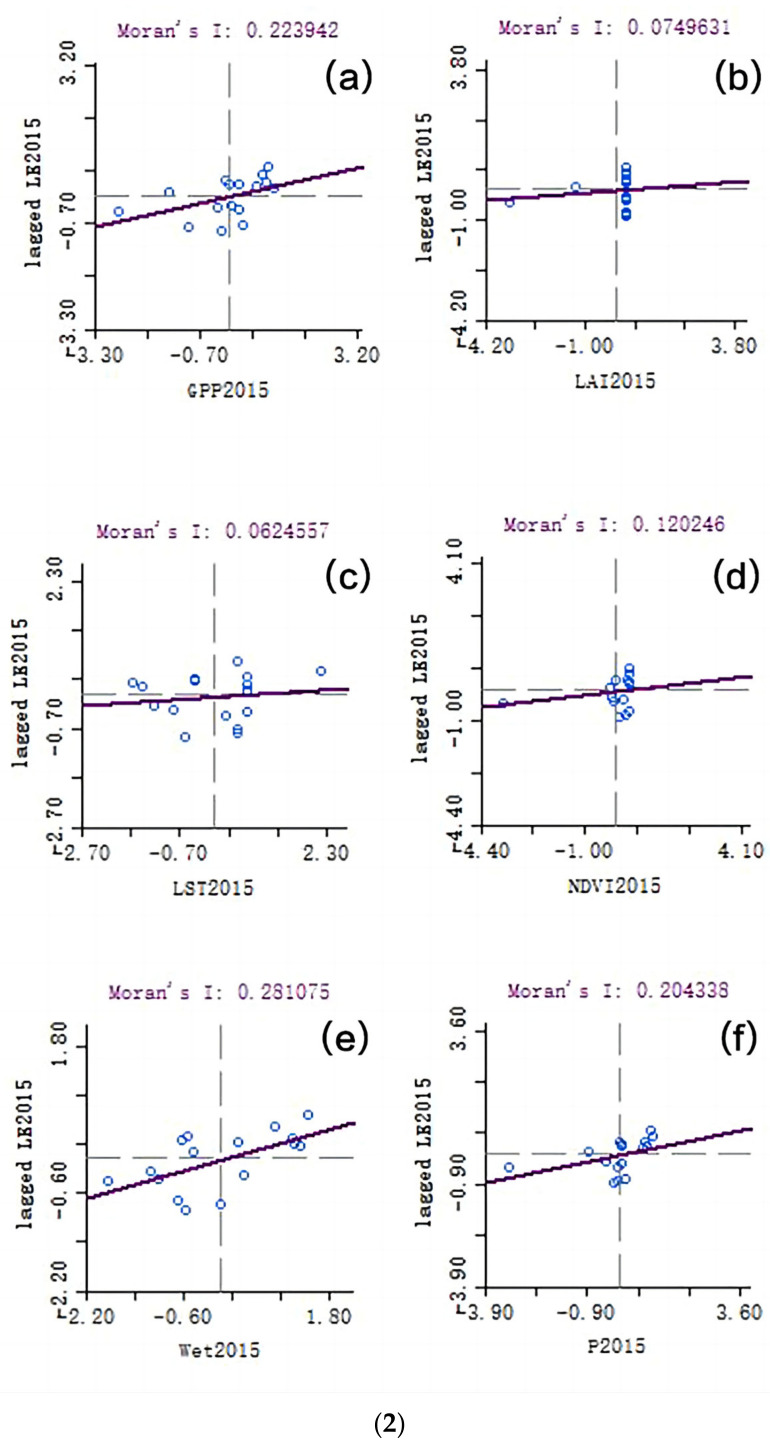

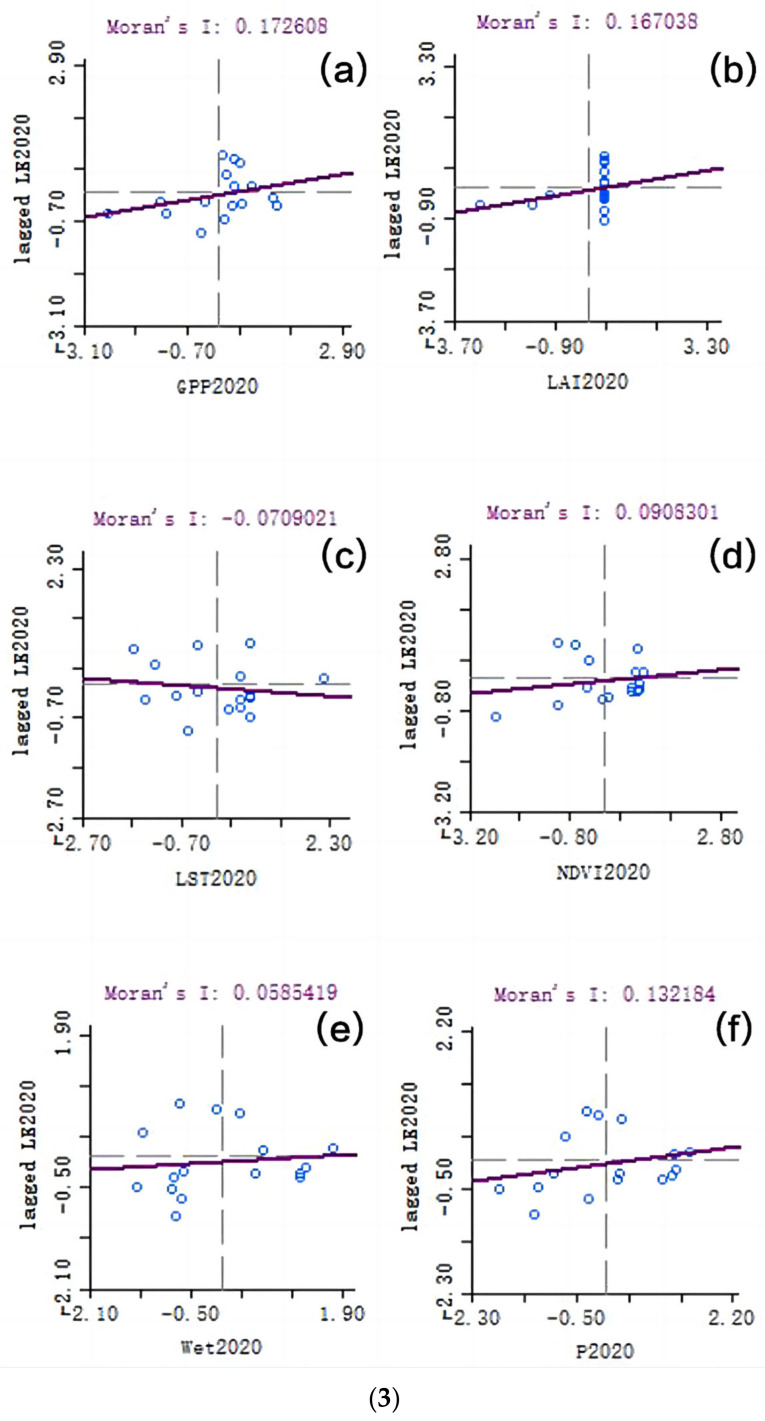
Results of spatial crosscorrelation analysis between the indexes and life expectancy (LE). (**1**) Results of spatial correlation analysis between the indexes and LE in 2010; (**a**) the Moran’s I results for life expectancy and gross primary production (GPP) in 2010; (**b**) the Moran’s I results for life expectancy and leaf area index (LAI) in 2010; (**c**) the Moran’s I results for life expectancy and land surface temperature (LST) in 2010; (**d**) the Moran’s I results for life expectancy and normalized difference vegetation index (NDVI) in 2010; (**e**) the Moran’s I results for life expectancy and Wet in 2010; (**f**) the Moran’s I results for life expectancy and comprehensive ecological index (P) in 2010. (**2**) Results of spatial correlation analysis between the indexes and LE in 2015, (**a**) the Moran’s I results for life expectancy and GPP in 2015; (**b**) the Moran’s I results for life expectancy and LAI in 2015; (**c**) the Moran’s I results for life expectancy and LST in 2015; (**d**) the Moran’s I results for life expectancy and NDVI in 2015; (**e**) the Moran’s I results for life expectancy and Wet in 2015; (**f**) the Moran’s I results for life expectancy and comprehensive ecological index in 2015. (**3**) Results of spatial correlation analysis between the indexes and LE in 2020, (**a**) The Moran’s I results for life expectancy and GPP in 2020; (**b**) the Moran’s I results for life expectancy and LAI in 2020; (**c**) the Moran’s I results for life expectancy and LST in 2020; (**d**) the Moran’s I results for life expectancy and NDVI in 2020; (**e**) the Moran’s I results for life expectancy and Wet in 2020; (**f**) the Moran’s I results for life expectancy and comprehensive ecological index in 2020.

GeoDa software was used to analyze the spatial crosscorrelation of the LE data and the various index data, and the Moran’s I results are shown in Figure 6. The data of the six indicators in 2010 and 2015 are positively correlated with the LE data. CEI in 2010 has the largest correlation with LE, while Wet (in 2015) has the largest correlation with LE. In 2020, except for the fact that LST is negatively correlated with LE, the other indicators are positively correlated with LE, and LAI has the largest correlation with LE. It can be seen from the figure that the LAI values of each city are mostly similar each year and are arranged in vertical lines.

The results of the Moran scatter diagrams in Figure 6 show the spatial crosscorrelation results of the annual LE data and various ecological indexes. The Moran’s I of the CEI in 2010 is about 0.228, which is the highest, indicating that the spatial crosscorrelation between the CEI and LE is the highest. This is consistent with the correlation coefficient results, where the Pearson coefficient of CEI is about 0.769, which is the highest; the Moran’s I for GPP in 2015 is about 0.224, which is second only to the Moran’s I of Wet, i.e., 0.281, indicating that the spatial crosscorrelation between GPP and LE is high. It is consistent with the correlation coefficient results, where the Pearson coefficient for GPP is about 0.653, which is the highest. The Moran’s I for GPP in 2020 is about 0.173, which is the highest, indicating that the spatial crosscorrelation between GPP and LE is the highest. This is consistent with the correlation coefficient results, where the Pearson coefficient for GPP is about 0.580, which is the highest. In conclusion, the spatial crosscorrelation between GPP and LE is higher than other ecological indicators.

##### Spatiotemporal Lag Spatial Crosscorrelation Analysis

**Figure 7:**
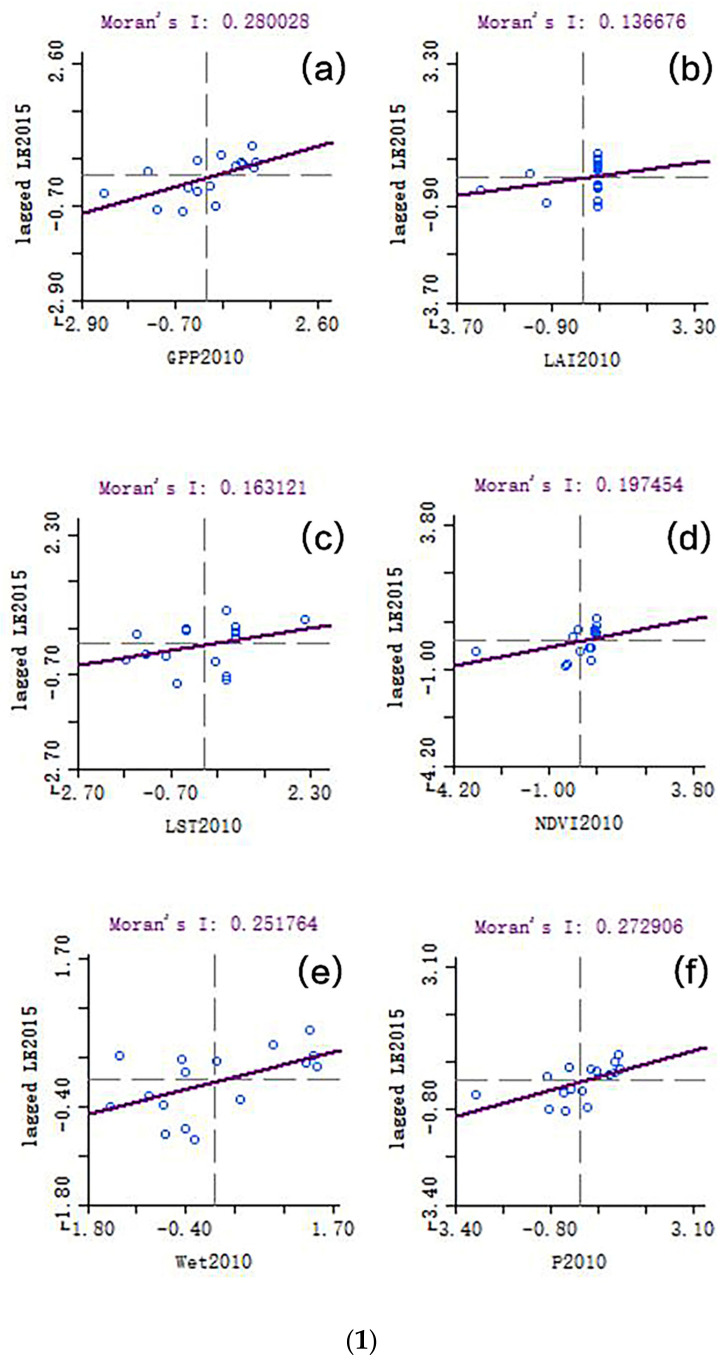

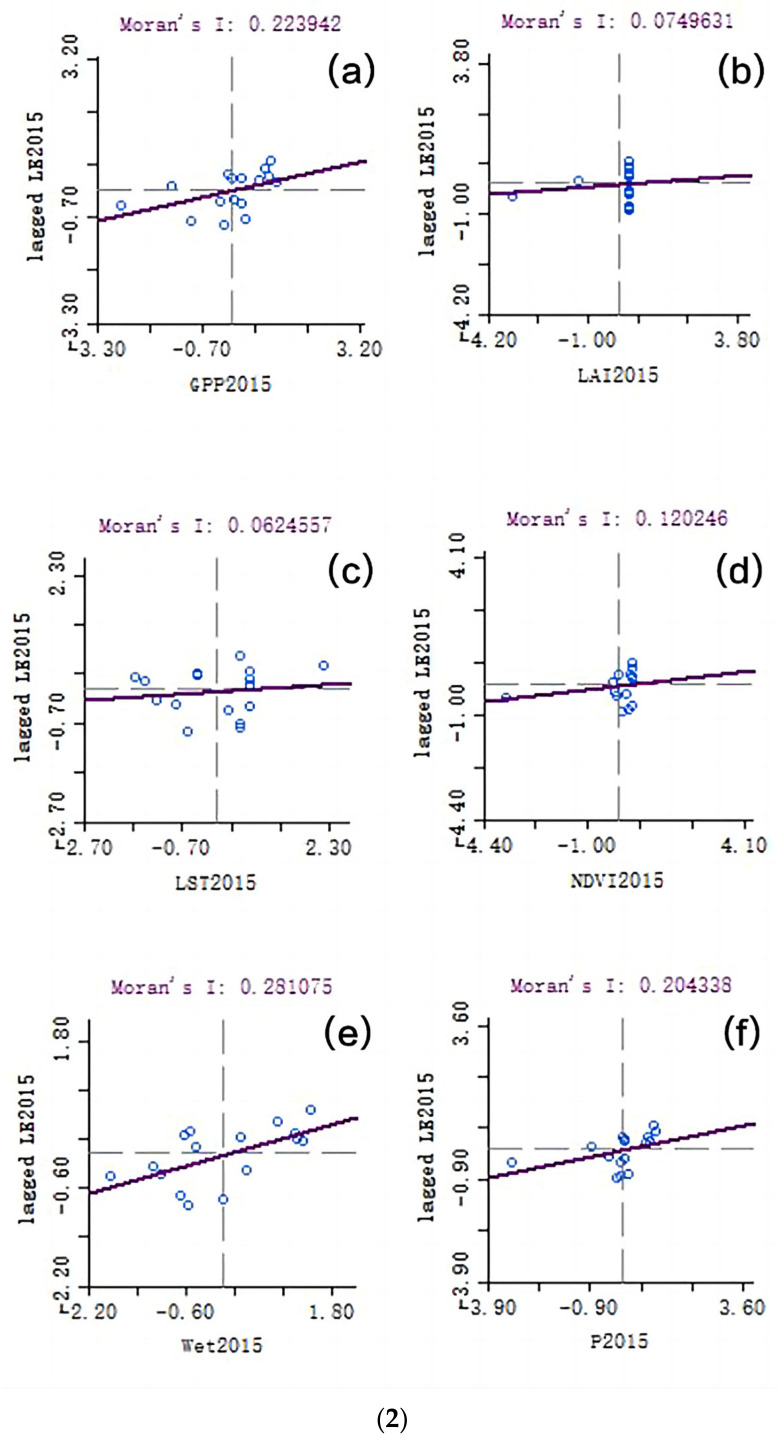

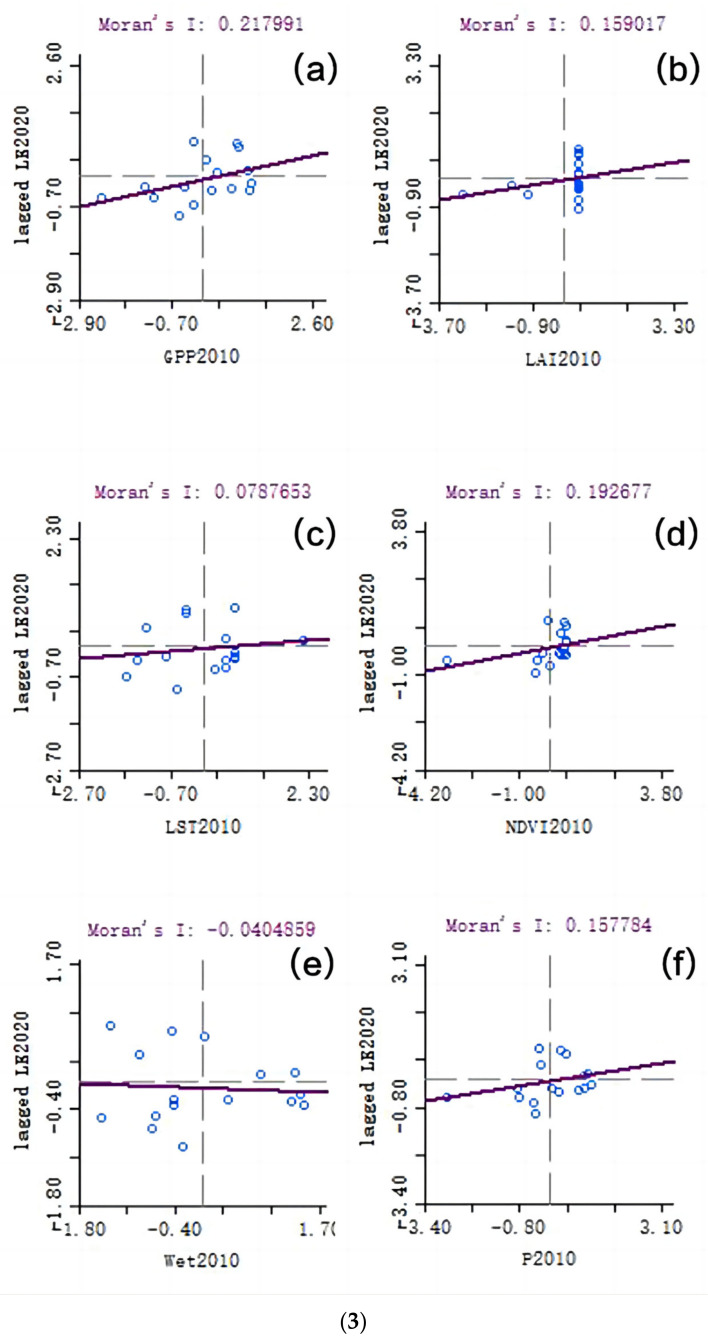
Results of spatiotemporal lag spatial crosscorrelation analysis between indicators and life expectancy (LE). (**1**) Analysis results of spatial lag between ecological indexes in 2010 and LE in 2015; (**a**) 2015 life expectancy and 2010 gross primary production (GPP) Moran’s I results; (**b**) 2015 life expectancy and 2010 leaf area index (LAI) Moran’s I results; (**c**) 2015 life expectancy and 2010 land surface temperature (LST) Moran’s I results; (**d**) 2015 life expectancy and 2010 normalized difference vegetation index (NDVI) Moran’s I results; (**e**) 2015 life expectancy and 2010 Wet Moran’s I results; (**f**) 2015 life expectancy and 2010 comprehensive ecological index (P) Moran’s I results. (**2**) Analysis results of spatial lag between ecological indexes in 2015 and LE in 2020; (**a**) 2020 life expectancy and 2015 GPP Moran’s I results; (**b**) 2020 life expectancy and 2015 LAI Moran’s I results; (**c**) 2020 life expectancy and 2015 LST Moran’s I result; (**d**) 2020 life expectancy and 2015 NDVI Moran’s I results, (**e**) 2020 life expectancy and 2015 Wet Moran’s I results; (**f**) 2020 life expectancy and 2015 comprehensive ecological index Moran’s I results. (**3**) Analysis results of spatial lag between ecological indexes in 2010 and LE in 2020; (**a**) 2020 life expectancy and 2010 GPP Moran’s I results; (**b**) 2020 life expectancy and 2010 LAI Moran’s I results; (**c**) 2020 life expectancy and 2010 LST Moran’s I results; (**d**) 2020 life expectancy and 2010 NDVI Moran’s I results; (**e**) 2020 life expectancy and 2010 Wet Moran’s I results; (**f**) 2020 life expectancy and 2010 comprehensive ecological index Moran’s I results.

GeoDa software was used to analyze the spatiotemporal lag spatial crosscorrelation between the LE data and the various index data. The Moran’s I results are shown in Figure 7. The data of each indicator in 2010 and 2015 are spatially correlated with the data of LE five years later (Figure 7 (1)a,b), and the data for each indicator in 2010 are spatially correlated with the data of LE after 2020 (Figure 7 (1)c). It can be seen from the results that the lag in the indicators in 2010 is positively correlated with the results for LE in 2015, and the correlation between NDVI and CEI is larger. As seen in Figure 7 (2), the lag of various indicator data in 2015 and the results obtained by the LE data in 2020 show that, except for the fact that LST is negatively correlated with LE, the rest show a positive correlation, and the correlation is small. As seen in Figure 7 (3), the spatial lag analysis results of various ecological indicators in 2010 and LE in 2020 show that, except for the fact that Wet is negatively correlated with LE, the rest are positively correlated, and GPP and NDVI have a strong correlation with LE.

The results of the Moran scatter diagram in Figure 7 show the results of the spatiotemporal lag spatial crosscorrelation analysis between the LE data and the various index data. The Moran’s I for GPP in 2010 and LE in 2015 is about 0.280, which is the highest, indicating that the spatiotemporal lag correlation between GPP and LE is the highest. It is inconsistent with the correlation coefficient results. When only considering the time lag, the Pearson coefficient for LAI is about 0.590, which is the highest; the Moran’s I of LAI in 2015 and LE in 2020 is about 0.124, which is the highest, followed by a Moran’s I for GPP in 2015 and LE in 2020, which is about 0.118. The Moran’s I for LST in 2015 and LE in 2020 is negative, which is about −0.071. This indicates that LAI and LE have the highest spatiotemporal lag correlation. In the lag correlation analysis (without considering spatial distribution), the Pearson coefficient for GPP is about 0.567, which is the highest, and the Moran’s I is inconsistent with the correlation coefficient; The Moran’s I for GPP in 2010 and LE in 2020 is about 0.218, which is the highest, indicating that the spatiotemporal lag correlation between GPP and LE is the highest. It is consistent with the correlation coefficient results. In the correlation analysis that only considers time lag, the Pearson coefficient for GPP is about 0.545, which is the highest. In conclusion, the spatiotemporal lag correlation between GPP and LE is higher than other ecological indicators.

**Figure 8:**
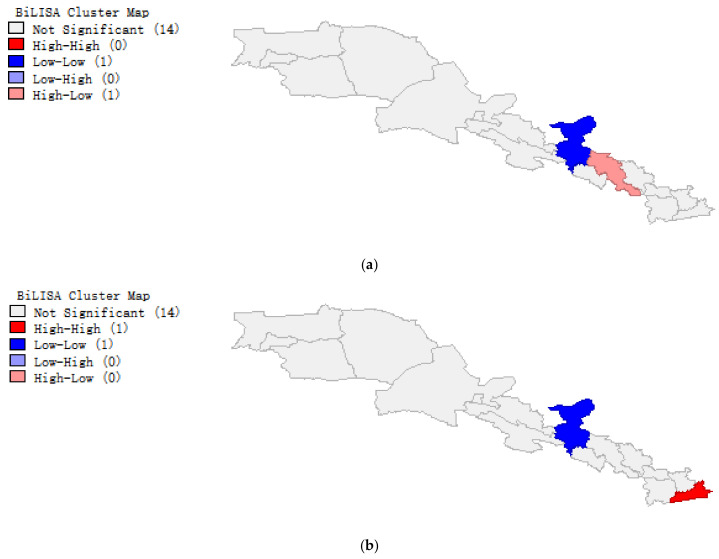

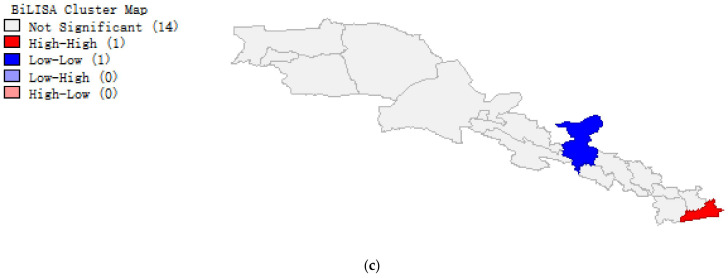
Cluster Map results. (**a**) Cluster Map of life expectancy (LE) in 2015 and comprehensive ecological index (CEI) in 2010. The figure shows low-low in Wuwei City, high-low in Baiyin City, and is not significant for the other cities. (**b**) Cluster Map of LE in 2020 and CEI in 2010. The figure shows low-low in Wuwei City and high-high in Xi’an City, and is not significant in the other cities. (**c**) Cluster Map of LE in 2020 and CEI in 2015. The figure shows low-low in Wuwei City and high-high in Xi’an City, and is not significant in the other cities.

The partial results of the Cluster Map obtained by GeoDa are shown in Figure 8. It can be seen that the LE in 2015 and the CEI in 2010 in the Cluster Map are low-low in Wuwei City, high-low in Baiyin City, and not significant in other cities; LE in 2020 and CEI in 2010, and LE in 2020 and CEI in 2015 in the Cluster Map show low-low in Wuwei City and high-high in Xi’an City.

### 4.3. Discussion

#### 4.3.1. Discussion of Correlation

This paper analyzes the correlation between six ecological indicators, i.e., NDVI, LAI, GPP, LST, Wet, and CEI, and the LE in 16 cities along the part of the “Silk Road Economic Belt” from Xi’an to Urumqi in Mainland China.

(1) Correlation coefficient

Longevity is the consequence of complex contributions. Without considering spatial distribution, the Pearson coefficients between each index and LE were calculated. The results show that GPP, LAI, Wet, NDVI, and *P* are positively related to LE, but the Pearson coefficient between LST and LE and the Pearson coefficient between Wet and LE are negative, which indicates that the impact of temperature and humidity is different from that of other indicators on LE. With an increase in temperature and humidity, LE decreases. In recent years, the intensification of climate change has made the temperature and humidity change correspondingly, which affects the change in LE. For example, the intensification of urban heat-island effects may aggravate the response to heat stress through an increase in outdoor temperature and humidity, thus affecting the health and LE of the residents [42].

(2) Spatial crosscorrelation

Spatial analysis is of great significance for the geographical phenomenon of public health inequality and the guidance of public health measures at the local scale [25]. The results of spatial crosscorrelation analysis on various indicators and LE shows that GPP, LAI, Wet, NDVI, and *P* are positively correlated with LE, but there are negative values for the Moran’s I for LST and Wet. It can be seen that the impact of temperature and humidity in adjacent cities on local LE is negatively correlated. The result of the cumulative greenhouse effect (year-by-year) on global warming needs to be reflected on a longer time scale, so LST in the study area has a small trend change from 2010 to 2020, but global warming is indisputably happening. Some studies show that appropriate temperature reduction is conducive to the extension of life span [43,44] and that the increase in temperature in adjacent cities may have a negative impact on the LE of local residents; the trend analysis results of Wet show that there are many areas with significant increases and decreases in the study area. The study area in this paper belongs to the northwest of China, with low humidity and drought. In recent decades, a “warm and wet” trend has been shown, and the humidity is gradually increasing. Besides, the improvement in humidity in adjacent cities may also affect the local LE.

(3) Consistency between Pearson coefficient and Moran’s I

The research results show that the Moran’s I value is basically consistent with the analysis results of the correlation coefficient, i.e., the Pearson coefficients and Moran’s I for GPP, LAI, Wet, NDVI, *P*, and LE are positive. The Moran’s I and Pearson coefficient between GPP and LE are relatively high, indicating that the correlation between GPP and LE is the highest. This result shows that there is a certain positive correlation between the ecological environment and LE, i.e., with an improvement in the quality of the ecological environment, LE also increases. In some of our experiments, the Pearson coefficient and Moran’s I between LST and LE are also consistent, i.e., they both have negative values, indicating that a rise in surface temperature has a negative impact on LE. Studies have shown that the occurrence of global warming may promote more frequent, more intense, and more lasting extreme temperature events, leading to an increase in heat-related mortality [15], which may affect LE. The Pearson coefficient and Moran’s I between Wet and LE also have negative values, indicating that humidity also has a certain impact on LE. In the research related to LE, the Wet index is rarely used as an influencing factor to link with LE. In the studies related to humidity, it has been found that humidity is associated with abnormal mortality and incidence rate levels through its role of influencing heat stress and hydration status [45], and its interaction with temperature will increase the incidence rate of cardiovascular and respiratory diseases [46], which may affect LE. 

In a word, the consistency between Moran’s I and the Pearson coefficients shows that the experimental results can be mutually verified, so the reliability of the experimental results has been proven.

#### 4.3.2. Discussion of Lag

The time lag for the response of LE to improvements in the ecological environment may weaken our understanding of the importance of the ecological environment on health [47]. The improvement of the ecological environment also has an indirect effect on LE in space. For example, the improvement of the environments in adjacent areas may promote the local LE, and the ecological environment may also have a lagging impact on health in space. Therefore, this study conducted spatiotemporal lag spatial crosscorrelation analysis on selected ecological indicators and LE. The results show that the Moran’s I obtained from the spatiotemporal lag analysis of the various ecological indicators in 2010 and LE in 2015 is the highest, but the Moran’s I obtained from the spatiotemporal lag analysis of the various ecological indicators in 2010 and LE in 2020 is higher than the Moran’s I obtained from the spatiotemporal lag analysis of the various ecological indicators in 2015 and LE in 2020. This result shows that the response of the life span health of people in the study area to the changes in ecological environment quality is relatively slow, and the lagging impact of the ecological environment on LE may need a long time scale to be reflected. Compared with Moran’s I obtained from the results of spatiotemporal lag spatial cross-correlation analysis, the Pearson coefficient between each index and LE obtained from simple time lag analysis is higher, i.e., the correlation is higher, indicating that the time lag impact of ecological environment between adjacent cities on LE of local residents is small. This may be caused by the lack of some data. Therefore, it is not clear in what time scale the impact of the ecological environment on life expectancy is the most intuitive and obvious.

#### 4.3.3. Discussion on the Influencing Factors

It can be seen from the experimental results that the optimization of the ecological environment has a positive impact on the growth of LE, but there are many other factors that also have a great impact on LE. For example, different spatial and temporal distributions of the geographical environment will also affect human health, which will lead to uneven distributions and the relative immobility of changes over time regarding LE [43]; the impact of the random occurrence of natural disasters on social population, economy, and mental health makes LE change [48,49]; the rapid development of the economy and society makes people pay more attention to their own health and promotes the development of medical and health services. The improvement of public health makes people’s LE increase gradually. There are also studies showing that regions with a more equal income distribution and a more developed economy have a more positive impact on LE [50], and the guarantee of national policies, such as pensions [51], medical insurance, and environmental protection, has a beneficial impact on the increase of LE.

## 5. Conclusions

This paper takes the area from Xi’an to Urumqi along the B&R as the research area. Based on MODIS product data and LE data from each city, this paper analyzes the relationship between the ecological environment and LE using PCA, trend analysis, cold and hot spot analysis, spatiotemporal lag spatial crosscorrelation analysis, and other methods and draws the following conclusions:

(1) Environmental policies in recent years have contributed to the improvement of the ecological environment of the cities along the B&R. There is a positive correlation between LE and the ecological environment of cities from Xi’an to Urumqi along the B&R. The PCA method was used to calculate the correlation between ecological indicators and LE, and more comprehensive results were obtained by combining time and space for the lag analysis;
(2) There is a lagging impact from the ecological environment on LE. The impact of ecological indicators in 2010 on LE in 2020 is greater than that in 2015. Therefore, the benefits of improving the ecological environment on LE may require a long time;
(3) In the correlation analysis between the ecological indicators and LE, GPP had the highest impact, which is beneficial to the growth of LE. However, LST and Wet have a negative impact on LE, which may be unfavorable to the growth of LE;

Future work will be carried out in the following two aspects:

(1) It is expected that this study can provide a new research idea for the related research of LE in other regions, so other regions along the B&R (e.g., abroad) will be selected for research;
(2) In this study, only five ecological indicators were selected, and CEI data were calculated. In the future, ecological indicators might be more accurate, making the experimental results better in order to provide some scientific reference for the government and relevant policies and suggestions. It is hoped that people can actively respond to the “double carbon” policy of carbon peaking and carbon neutrality, control carbon emissions to slow down global warming, and promote the healthy development of human society.

## Figures and Tables

**Table 1:** Research indicators and data products.

Indicator	Product	Time Resolution (Day)	Space Resolution (m)	Grade
NDVI	MOD13Q1	16	250	L3
GPP	MOD17A2H.	8	500	L4
LAI	MOD15A2H	8	500	L4
LST	MOD11A2	8	1000	L3
Wet	MOD09A1 (Surface reflectance)	8	500	L3

**Table 2:** Statistical table of indicators.

	Number of Pixels	Observations	Mean	Standard Deviations	Minimum	Maximum
GPP2010	13,555,431(4523, 2997)	736	0.013162	0.018770	0.000000	0.101799
GPP2015	13,555,431(4523, 2997)	720	0.012964	0.018558	0.000000	0.101599
GPP2020	13,555,431(4523, 2997)	736	0.012224	0.017662	0.000000	0.103699
LAI2010	8,175,867(4417, 1851)	736	0.686858	1.284290	0.000000	7.000000
LAI2015	8,175,867(4417, 1851)	736	0.752034	1.449256	0.000000	7.000000
LAI2020	8,175,867(4417, 1851)	736	0.721788	1.423109	0.000000	7.000000
LST2010	1,968,544(2168, 908)	736	45.612089	9.433674	0.000000	69.000000
LST2015	1,968,544(2168, 908)	736	45.612089	9.433674	0.000000	69.000000
LST2020	1,968,544(2168, 908)	736	45.606350	9.438040	0.000000	69.000000
NDVI2010	3,390,738(2262, 1499)	368	0.271135	0.240782	−0.183699	0.998699
NDVI2015	3,390,738(2262, 1499)	368	0.275488	0.247503	−0.163000	0.996899
NDVI2020	3,390,738(2262, 1499)	368	0.269964	0.242245	−0.188199	0.999399
WET2010	8,774,109(4577, 1917)	736	0.079296	0.196396	−0.607913	0.889902
WET2015	8,774,109(4577, 1917)	736	0.004665	0.235381	−0.997439	0.912903
WET2020	8,774,109(4577, 1917)	736	0.042862	0.197213	−0.863188	0.945436

**Table 3:** LE of cities or states along the Belt and Road.

Cities or Autonomous Prefectures along the Belt and Road	Life Expectancy (Years)		
	2010	2015	2020
Xi’an City	75.86	76.27	80.86
Baoji City	72.12	75.00	77.88
Xianyang City	72.00	74.50	77.00
Lanzhou City	72.54	73.50	74.50
Jiayuguan City	77.79	77.99	78.19
Baiyin City	72.84	74.00	75.16
Wuwei City	71.34	73.50	75.66
Zhangye City	70.41	72.30	74.20
Pingliang City	72.23	73.25	74.00
Jiuquan City	72.81	74.80	75.60
Haibei Tibetan Autonomous Prefecture	70.41	71.33	72.24
Urumqi City	73.70	75.80	76.40
Turpan City	71.40	73.60	74.35
Hami City	74.00	75.00	76.00
Changji Hui Autonomous Prefecture	73.25	75.49	76.16
Guyuan City	70.45	71.85	76.80

**Table 4:** The contribution rates and characteristic values of the principal components.

Indexes	2010		2015		2020		
	PC1	PC2	PC1	PC2	PC1	PC2	PC3
GPP	0.955	0.008	0.930	0.048	0.941	−0.215	0.151
LAI	0.912	−0.219	0.931	−0.079	0.795	−0.458	0.344
LST	−0.072	0.955	−0.113	0.984	−0.254	0.560	0.787
NDVI	0.933	−0.030	0.941	0.002	0.546	0.709	−0.331
Wet	0.744	0.387	0.763	0.182	0.826	0.389	−0.042
Eigenvalue	3.172	1.111	3.213	1.009	2.564	1.224	0.872
Contribution rate of variance (%)	63.446	22.217	64.262	20.183	51.272	24.486	17.448
Accumulated variance contribution rate (%)	63.446	85.663	64.262	84.445	51.272	75.758	93.206

**Table 5:** Correlation analysis between indexes and LE.

Correlation Index	2010LE	2015LE	2020LE
2010 GPP	0.732193	0.346175	0.545161
2010 LAI	0.521710	0.589989	0.545020
2010 LST	0.070000	−0.311609	−0.158746
2010 NDVI	0.702664	0.199525	0.356853
2010 Wet	0.672466	−0.209284	−0.148997
2010 CEI(P)	0.769155	0.234094	0.388458
2015 GPP	—	0.652993	0.566851
2015 LAI	—	0.326956	0.492288
2015 LST	—	−0.268887	−0.534790
2015 NDVI	—	0.594811	0.277257
2015 Wet	—	0.432666	−0.291204
2015 CEI(P)	—	0.535910	0.252389
2020 GPP	—	—	0.579655
2020 LAI	—	—	0.544977
2020 LST	—	—	−0.170294
2020 NDVI	—	—	−0.264197
2020 Wet	—	—	0.177764
2020 CEI(P)	—	—	0.346410

## Data Availability

Most of the datasets used in this study are publicly available and can be downloaded after signing a Data Usage Agreement. The MODIS dataset is available at https://ladsweb.modaps.eosdis.nasa.gov/ (accessed on 7 December 2022); The life expectancy dataset was obtained from official release documents of each city.

## References

[1] Ascensão F., Fahrig L., Clevenger A.P., Corlett R.T., Jaeger JA G., Laurance W.F., Pereira H.M. (2018). Environmental challenges for the Belt and Road Initiative. Nat. Sustain..

[2] Huang Y. (2022). The Health Silk Road: How China Adapts the Belt and Road Initiative to the COVID-19 Pandemic. Am. J. Public Health.

[3] Ferdinand P. (2016). Westward ho—The China dream and ‘one belt, one road’: Chinese foreign policy under Xi Jinping. Int. Aff..

[4] Huang Y. (2016). Understanding China’s Belt & Road initiative: Motivation, framework and assessment. China Econ. Rev..

[5] Kjellstrom T., Holmer I., Lemke B. (2009). Workplace heat stress, health and productivity–an increasing challenge for low and middle-income countries during climate change. Glob. Health Action.

[6] Loeppke R., Taitel M., Haufle V., Parry T., Kessler R.C., Jinnett K. (2009). Health and productivity as a business strategy: A multiemployer study. J. Occup. Environ. Med..

[7] Hu R., Liu R., Hu N. (2017). China’s Belt and Road Initiative from a global health perspective. Lancet Glob. Health.

[8] Chen H., Wang J., Yu X., Li C., Zhao Y., Xing Y., Li X. (2022). Policies of voluntary services involved in public health emergencies in China: Evolution, evaluation, and expectation. Front. Public Health.

[9] Yang T., Zhou K., Ding T. (2022). Air pollution impacts on public health: Evidence from 110 cities in Yangtze River Economic Belt of China. Sci. Total Environ..

[10] Patz J.A., Campbell-Lendrum D., Holloway T., Foley J.A. (2005). Impact of regional climate change on human health. Nature.

[11] Li L., Zhang Y., Zhou T., Wang K., Wang C., Wang T., Lü G. (2022). Mitigation of China’s carbon neutrality to global warming. Nat. Commun..

[12] Liu Z., Deng Z., He G., Wang H., Zhang X., Lin J., Liang X. (2022). Challenges and opportunities for carbon neutrality in China. Nat. Rev. Earth Environ..

[13] Zhao X., Ma X., Chen B., Shang Y., Song M. (2022). Challenges toward carbon neutrality in China: Strategies and countermeasures. Resour. Conserv. Recycl..

[14] Landsea C.W. (2005). Hurricanes and global warming. Nature.

[15] Wang Y., Wang A., Zhai J., Tao H., Jiang T., Su B., Gao C. (2019). Tens of thousands additional deaths annually in cities of China between 1.5 C and 2.0 C warming. Nat. Commun..

[16] Laghari J. (2013). Climate change: Melting glaciers bring energy uncertainty. Nature.

[17] Wunderling N., Willeit M., Donges J.F., Winkelmann R. (2020). Global warming due to loss of large ice masses and Arctic summer sea ice. Nat. Commun..

[18] Oeppen J., Vaupel J.W. (2002). Broken limits to life expectancy. Science.

[19] Wang S., Ren Z., Liu X., Yin Q. (2022). Spatiotemporal trends in life expectancy and impacts of economic growth and air pollution in 134 countries: A Bayesian modeling study. Soc. Sci. Med..

[20] Gulis G. (2000). Life expectancy as an indicator of environmental health. Eur. J. Epidemiol..

[21] Nkalu C.N., Edeme R.K. (2019). Environmental hazards and life expectancy in Africa: Evidence from GARCH model. Sage Open.

[22] Wu Y., Wang W., Liu C., Chen R., Kan H. (2020). The association between long-term fine particulate air pollution and life expectancy in China, 2013 to 2017. Sci. Total Environ..

[23] Cheng J., Ho H.C., Webster C., Su H., Pan H., Zheng H., Xu Z. (2021). Lower-than-standard particulate matter air pollution reduced life expectancy in Hong Kong: A time-series analysis of 8.5 million years of life lost. Chemosphere.

[24] Gebremichael M., Barros A.P. (2006). Evaluation of MODIS Gross Primary Productivity (GPP) in tropical monsoon regions. Remote Sens. Environ..

[25] Ladoy A., Vallarta-Robledo J.R., De Ridder D., Sandoval J.L., Stringhini S., Da Costa H., Joost S. (2021). Geographic footprints of life expectancy inequalities in the state of Geneva, Switzerland. Sci. Rep..

[26] Liu W., Xu Z., Yang T. (2018). Health effects of air pollution in China. Int. J. Environ. Res. Public Health.

[27] Xiao R., Guo D., Ali A., Mi S., Liu T., Ren C., Zhang Z. (2019). Accumulation, ecological-health risks assessment, and source apportionment of heavy metals in paddy soils: A case study in Hanzhong, Shaanxi, China. Environ. Pollut..

[28] Zhang X., Yang L., Li Y., Li H., Wang W., Ye B. (2012). Impacts of lead/zinc mining and smelting on the environment and human health in China. Environ. Monit. Assess..

[29] Caçador I., Neto J., Duarte B., Barroso D., Pinto M., Marques J. (2013). Development of an Angiosperm Quality Assessment Index (AQuA-Index) for ecological quality evaluation of Portuguese water bodies—A multi-metric approach. Ecol. Indic..

[30] Li J., Song C., Cao L., Zhu F., Meng X., Wu J. (2011). Impacts of landscape structure on surface urban heat islands: A case study of Shanghai, China. Remote Sens. Environ..

[31] An M., Xie P., He W., Wang B., Huang J., Khanal R. (2022). Spatiotemporal change of ecologic environment quality and human interaction factors in three gorges ecologic economic corridor, based on RSEI. Ecol. Indic..

[32] Hu X., Xu H. (2018). A new remote sensing index for assessing the spatial heterogeneity in urban ecological quality: A case from Fuzhou City, China. Ecol. Indic..

[33] Zhang J., Zhu Y., Fan F. (2016). Mapping and evaluation of landscape ecological status using geographic indices extracted from remote sensing imagery of the Pearl River Delta, China, between 1998 and 2008. Environ. Earth Sci..

[34] Ouyang X., Wang J., Chen X., Zhao X., Ye H., Watson A.E., Wang S. (2021). Applying a projection pursuit model for evaluation of ecological quality in Jiangxi Province, China. Ecol. Indic..

[35] Li Z.-W., Zeng G.-M., Zhang H., Yang B., Jiao S. (2007). The integrated eco-environment assessment of the red soil hilly region based on GIS—A case study in Changsha City, China. Ecol. Model..

[36] Tucker C.J. (1979). Red and photographic infrared linear combinations for monitoring vegetation. Remote Sens. Environ..

[37] Knyazikhin Y., Martonchik J., Myneni R.B., Diner D., Running S.W. (1998). Synergistic algorithm for estimating vegetation canopy leaf area index and fraction of absorbed photosynthetically active radiation from MODIS and MISR data. J. Geophys. Res. Atmos..

[38] Running S.W., Nemani R.R., Heinsch F.A., Zhao M., Reeves M., Hashimoto H. (2004). A continuous satellite-derived measure of global terrestrial primary production. Bioscience.

[39] Benali A., Carvalho A., Nunes J., Carvalhais N., Santos A. (2012). Estimating air surface temperature in Portugal using MODIS LST data. Remote Sens. Environ..

[40] Wan Z. (2007). Collection-5 MODIS Land Surface Temperature Products Users’ Guide.

[41] Lobser S., Cohen W. (2007). MODIS tasselled cap: Land cover characteristics expressed through transformed MODIS data. Int. J. Remote Sens..

[42] Tan J., Zheng Y., Tang X., Guo C., Li L., Song G., Li F. (2010). The urban heat island and its impact on heat waves and human health in Shanghai. Int. J. Biometeorol..

[43] Wang S., Luo K., Liu Y. (2015). Spatio-temporal distribution of human lifespan in China. Sci. Rep..

[44] Conti B. (2008). Considerations on temperature, longevity and aging. Cell. Mol. Life Sci..

[45] Davis R.E., McGregor G.R., Enfield K.B. (2016). Humidity: A review and primer on atmospheric moisture and human health. Environ. Res..

[46] Lin S., Luo M., Walker R.J., Liu X., Hwang S.-A., Chinery R. (2009). Extreme high temperatures and hospital admissions for respiratory and cardiovascular diseases. Epidemiology.

[47] Watts K., Whytock R.C., Park K.J., Fuentes-Montemayor E., Macgregor N.A., Duffield S., McGowan P.J. (2020). Ecological time lags and the journey towards conservation success. Nat. Ecol. Evol..

[48] Neumayer E., Plümper T. (2007). The gendered nature of natural disasters: The impact of catastrophic events on the gender gap in life expectancy, 1981–2002. Ann. Assoc. Am. Geogr..

[49] Noy I. (2016). Natural disasters in the Pacific Island Countries: New measurements of impacts. Nat. Hazards.

[50] Wilkinson R.G. (1992). Income distribution and life expectancy. BMJ Br. Med. J..

[51] Bravo J.M., Ayuso M., Holzmann R., Palmer E. (2021). Addressing the life expectancy gap in pension policy. Insur. Math. Econ..

